# Evaluation of caffeine modulation of topiramate effect on locomotor activity of zebrafish larvae in pentylenetetrazol-induced seizure model

**DOI:** 10.1371/journal.pone.0317241

**Published:** 2025-03-04

**Authors:** Adrian Bartoszek, Emilia Fornal

**Affiliations:** Department of Bioanalytics, Medical University of Lublin, Lublin, Poland; Helwan University, EGYPT

## Abstract

Epilepsy is a prevalent neurological condition marked by seizures that lead to neurobiological and behavioral impairments. Caffeine (CAF), the world’s most consumed stimulant, reportedly affects both epileptic seizures and the efficacy of antiepileptic drugs, particularly topiramate (TPM). This study aimed to investigate the effects of CAF on TPM in a pentylenetetrazol (PTZ)-induced seizure model using zebrafish larvae. Four days post-fertilization *Danio rerio* larvae were incubated for 18 hours with CAF, TPM, or CAF+TPM, followed by an assessment of locomotor activity. Seizures were induced by adding PTZ to achieve a final concentration of 20 mM. In the PTZ-induced seizure model, the application of CAF in doses over 50 mg/L resulted in a decrease in the average movement. TPM ( > 50 μM) significantly protected larvae against the PTZ. The addition of 15 mg/L CAF to TPM did not affect larval activity at any TPM concentration tested; however, higher doses of CAF significantly reduced larval activity. CAF doses above 25 mg/L altered the activity of larvae treated with TPM in the PTZ-induced seizure model. Larvae exhibited differential heart rate (HR) responses to CAF exposure across doses. CAF at 75 mg/L significantly increased HR, while doses of 175 mg/L and higher induced bradycardia. TPM, across all tested doses, did not independently influence HR. The study provides valuable insights into the interactions between CAF and TPM, which may inform future research on human epilepsy. However, the extrapolation of these results to other species should be approached cautiously due to physiological differences.

## Introduction

Epilepsy, a prevalent neurological disorder characterized by seizures leading to neurobiological and behavioral impairments, ranks as the second most common neurological condition following migraine, affecting 1-2% of the global population [1]. This condition not only leads to physical complications such as bruises and fractures following seizures but also induces significant psychological distress, including anxiety and depression [1]. Despite the existence of over 30 antiepileptic drugs, there is no cure for epilepsy; treatments are merely symptomatic [2]. Approximately one-third of those with epilepsy do not respond to these medications [3]. Furthermore, there are no preventive treatments for those at risk, even though our understanding of the underlying molecular and cellular pathways has grown. The complexity and heterogeneity of epilepsy significantly hinder progress in these areas [1].

Caffeine (CAF), a member of the purine alkaloid group, is the most extensively consumed stimulant globally. About 80% of the world’s population consumes CAF-infused beverages, with North America and Europe having the highest per capita consumption [4,5]. The average daily consumption from sources like coffee, tea, and soft drinks is approximately 300 mg per person, considered a pharmacologically active dose [4]. CAF not only impacts epileptic seizures but also affects the efficacy of anticonvulsant drugs, complicating the management of seizures [5].

The interaction between CAF and epilepsy is poorly understood, with limited clinical data primarily sourced from preclinical animal studies, a handful of clinical trials, and some case studies [6,7]. These studies suggest a complex and not yet fully understood relationship between epileptic seizures, CAF, and antiepileptic drugs, thereby complicating the establishment of clear clinical guidelines for the intake of CAF among individuals with or at risk for epilepsy. Evidence from preclinical studies suggests that CAF may increase susceptibility to seizures, yet in some scenarios, long-term intake could confer protective effects against seizures. Additionally, CAF has been shown to diminish the effectiveness of several antiepileptic drugs, with the most significant impact noted with topiramate (TPM) [6,7].

Regarding research models, the zebrafish (*Danio rerio*) has emerged over the past two decades as a valuable *in vivo* model due to its 70% genetic homology with humans, which is utilized across various stages of research [8]. This species offers advantages such as low maintenance costs, rapid development, and minimal ethical concerns, making it a preferred model in neurological studies [9]. In light of the principle of the three R (Replacement, Reduction, and Refinement), larvae provide a balanced compromise for scientific research [10]. By employing zebrafish larvae, researchers can minimize the number of animals required while still obtaining valuable insights into biological processes, thereby enhancing the ethical framework of animal research [9].

The central nervous system of zebrafish exhibits a high degree of homology with humans, providing a robust basis for extrapolating results from zebrafish to human studies [11]. It was presented that the dynamics of seizures in zebrafish and humans are remarkably similar, which enhances the transferability of results from the animal to humans [12]. Zebrafish are well-known animal model to effectively indicate potential drug toxicity to the human cardiovascular system [13]. Research indicates that human cardiac electrophysiology closely resembles zebrafish, more so than rodents [14]. The zebrafish heart, with its two chambers—an atrium and a ventricle—shares key similarities with mammalian hearts at cellular and molecular levels [15]. Blood flows from the sinus venosus to the atrium, through the ventricle, and into the aorta, regulated by valves. Its heart rhythm and contractions are controlled by electrical conduction, including a pacemaker system, paralleling mammalian mechanisms [16]. *Danio rerio* has a heart rate (HR) of 140–180 beats per minute (BPM), comparable to the human fetal HR (130–170 bpm) and significantly slower than the mouse (300–600 BPM) [17]. A previous study found that over 95% of drugs causing QT prolongation in humans also produce similar effects in zebrafish [18]. Zebrafish larvae, serving as an intermediate between *in vitro* and *in vivo* mammal studies, enable comprehensive examination within a whole vertebrate organism.

Various chemical and genetic models are utilized according to the specific research objectives concerning seizures, with the pentylenetetrazol (PTZ)-induced seizure model recognized as one of the most thoroughly documented chemical models in both zebrafish and rodent species [19]. The interaction of CAF on TPM in chemically induced seizures has been explored in various animal models but has not been sufficiently studied in zebrafish [7]. The outcomes of these studies are mixed and necessitate further investigation to elucidate the mechanisms involved and to determine the most suitable models for future research. The PTZ model was adopted for zebrafish in 2005 and has since been validated in numerous studies concerning antiepileptic drugs [20]. Afrikanova et al. highlighted the anticonvulsant activity of TPM against PTZ-induced seizures (20 mM), prompting the use of this model in our study [13].

The aim of the study was to evaluate the influence of CAF on TPM in the pentylenetetrazol-induced seizure model in zebrafish larvae. The locomotor activity of zebrafish larvae was studied to examine the effect of CAF and TPM interaction.

## Materials and methods

### Animals

*Danio rerio* stocks of the wild type zebrafish strain (AB strain, Experimental Medicine Centre, Medical University of Lublin, Poland) were maintained at a temperature of 26–28.5 °C in a controlled environment (pH ranging between 6.9 and 7.5; conductivity of 550–700; 14/10 h light/dark cycle). Embryos were reared under a standard light/day cycle in an E3 embryo medium in an incubator (IN 110 Memmert GmbH, Buechenbach, Germany). The 4 days post-fertilization (dpf) zebrafish larvae were used for the assays. After the experiment, larvae were immediately killed by immersion in a solution of tricaine (1,6 g/L) [21]. All experiments were conducted in accordance with the National Institute of Health Guidelines for the Care and Use of Laboratory Animals and the European Community Council Directive for the Care and Use of Laboratory Animals of 22 September 2010 (2010/63/EU). For the experiment with larvae up to 5 dpf, agreement with the Local Ethical Commission is not required.

### Chemicals

Topiramate (97240-79-4), caffeine (58-08-2), pentylenetetrazol (54-95-5) and methylcellulose (9004-67-5) were purchased from Sigma-Aldrich (Saint Louis, MI, USA). All compounds were dissolved in deionized water and diluted in E3 embryo medium (pH 7.1–7.3; 17.4 μM NaCl, 0.21 μM KCl, 0.12 μM MgSO_4_ and 0.18 μM Ca(NO_3_)_2_) to achieve a designated concentration.

### Toxicological evaluation

Initially, toxicological evaluations for both TPM and CAF were conducted. The concentrations were chosen based on the literature review [20,22]. For that, 4 dpf zebrafish larvae were incubated for 18h at 28°C with the compounds (500 µ L of the solution to each well) at concentrations—25 mg/L, 50 mg/L, 75 mg/L, 100 mg/L, 125 mg/L, 150 mg/L, 175 mg/L, 200 mg/L, 225 mg/L, 250 mg/L, and 25 μM; 50 μM, 75 μM, 100 μM, 125 μM, 150 μM, 175 μM, 200 μM, 225 μM, for CAF and TPM respectively, as well as the combination of them (50 mg/L CAF and 75 μM TPM; 100 mg/L CAF and 75 μM TPM; 150 mg/L CAF and 200 μM T;, 200 mg/L CAF and 200 μM TPM) in 48 well-plates. Five larvae were placed in each of the wells, which gives a total of 15 larvae per concentration. As a control, an E3 solution was used. Larvae were assessed morphologically using a light microscope (Zeiss Stemi 508 microscope) throughout the experiment. The succeeding symptoms of acute locomotor impairment were estimated: hypoactivity (reduced; no touch response), loss of posture, body deformation (pigmentation changes, jaw malformations, body axis alteration), exophthalmos (eye protrusion), organ dysfunction (deflated swim bladder, pericardial edema), and finally dead [20,23]. The experiments were performed in triplicate.

### Heart rate assessment

HR was measured in five randomly selected larvae per treatment [24]. The animals were equilibrated at room temperature for 30 min and immobilised on 3% methylcellulose. The HRs were counted under a stereomicroscope (Zeiss Stemi 508 microscope) for 20 s manually. The obtained values were multiplied by three to obtain the HR per minute [24].

### Evaluation of locomotor behavior

**Fig 1:**
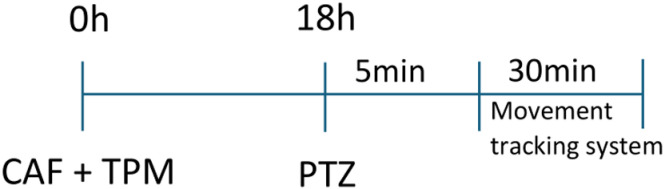
Schematic experimental timelines were used for the behavioral tracking.

Larvae were preincubated in 100 ml of E3 embryo medium or tested substances for 18 hours in individual wells of a 96-well plate at 28 °C (for timeline schematic, see Fig 1). The experiments were conducted in the afternoon since larvae behavior is more stable between noon and afternoon [25,26]. Ten larvae were used per treatment parameter and per experiment. The concentrations of CAF (5-100 mg/L) and TPM (25-175 μM) used in the study were chosen based on the toxicological evaluation of compounds. After the preincubation, 100 μl of E3 embryo medium or 100 μl of a 40 mM PTZ solution was added to obtain a final concentration of 20 mM to evoke seizures [20]. Larvae were allowed to habituate for 5 min in a dark chamber of an automated tracking device (ZebraBox^TM^ apparatus; Viewpoint, Lyon, France). The total locomotor activity was then quantified using ZebraLab^TM^ software (Viewpoint, Lyon, France) [20]. Average movement or activity was expressed in “actinteg” units. The actinteg value of the ZebraLab^TM^ software is defined as the sum of all image pixel changes detected during the time slice defined for the experiment (30 minutes). All tracking experiments were performed in triplicate.

### Statistical analysis

Statistical analyses were performed by GraphPad Prism 8 (GraphPad Software, San Diego, CA, USA). For comparison, data were analyzed using analysis of variance (one-way or two-way ANOVA). One-way ANOVA was followed by the Tukey’s test (post hoc test). In the case of two-way ANOVA, Bonferroni’s test was used as a post hoc test. The confidence limit of p < 0.05 was considered statistically significant. Data are presented as mean ± standard deviation (SD). Zebrafish larvae were randomly allocated to experimental groups.

## Results

### Toxicological evaluation and HR assessment

**Fig 2:**
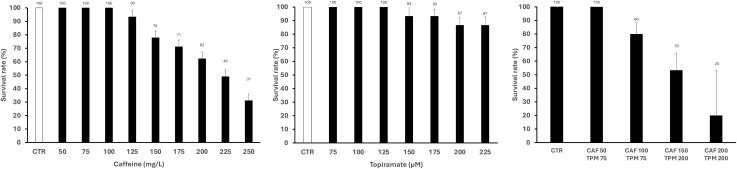
The survival rate of larvae treated with caffeine (CAF), topiramate (TPM), or combination.

For survival assessment, the animals were treated with CAF and TPM for 18 h (Fig 2). CAF immersion did not reduce survival rate, compared to controls, up to dose 100 mg/L, while 125 mg/L, 150 mg/L, 175 mg/L, 200 mg/L, 225 mg/L, and 250 mg/L decreased the survival rate to 93%, 78%, 71%, 62%, 49%, and 31% respectively. TPM incubation did not change the survival rate, compared to controls, up to 125 μM and the doses 150 μM, 175 μM, 200 μM, and 225 μM reduced it to 93%, 93%, 87%, and 87%, respectively. In compound combination, the survival rates stand as: 50 mg/L CAF in combination with 75 μM TPM- 100%; 100 mg/L CAF and 75 μM TPM - 80%, 150 mg/L CAF and 200 μM TPM - 53%, 200 mg/L CAF and 200 μM TPM- 20% (Fig 2).

**Fig 3:**
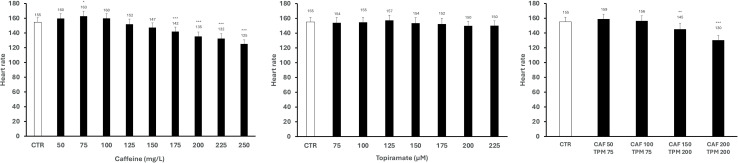
Heart rate of larvae treated with caffeine (CAF), topiramate (TPM), or combination; * p < 0.05, ** p < 0.01, *** p < 0.001, as compared to control.

Control larvae at 5 dpf exhibit 155 BPM (Fig 3). CAF in dose 75 mg/L increased the larvae HR compared to the control group, while doses 175 mg/L and more decreased it. TPM does not affect the HR in any investigated doses. When combined, the HR decreased when larvae were treated with 150 mg/L CAF and 200 μM TPM, 200 mg/L CAF, and 200 μM TPM (Fig 3).

### The influence of CAF on larval locomotor activity

**Fig 4:**
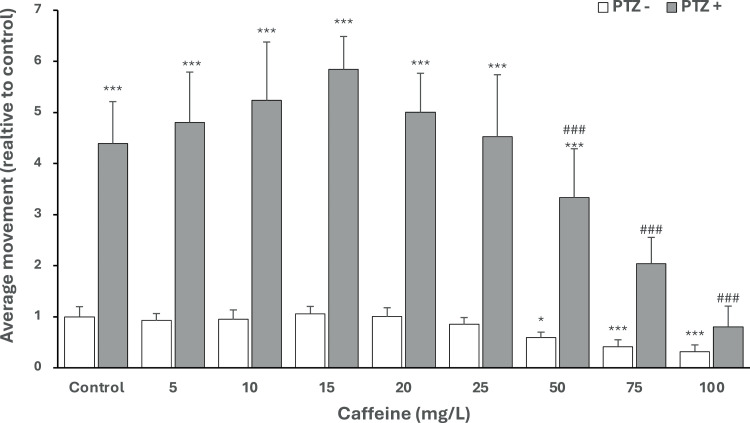
Effect of caffeine (CAF) on average larval locomotor movement without pentylenetetrazol (PTZ -), with PTZ-induced seizures (PTZ+ ); * p < 0.05, *** p < 0.001, as compared to control (PTZ-); ### p < 0.001 as compared to control (PTZ+); average movement is presented as referring to control (PTZ-); (1 = 29460 actinteg units).

Firstly, the average movement of zebrafish larvae, expressed in “actinteg” units, under the influence of CAF (concentration range of 5 to 100 mg/L) was assessed. After 18h of CAF incubation at 50 mg/L, 75 mg/L, and 100 mg/L dilutions, a decrease in the average movement was observed, as shown in Fig 4. At lower CAF concentrations, no statistically significant change in locomotor activity was observed, yet a slight increase in the activity is noticeable. In the PTZ-induced seizure model, the application of CAF in doses 50 mg/L, 75 mg/L, and 100 mg/L resulted in a decrease of the average movement in comparison to PTZ group, and zebrafish treated with 75mg/L and 100mg/L CAF showed the activity at the level of control animals (Fig 4).

### The influence of TPM on larval locomotor activity

**Fig 5:**
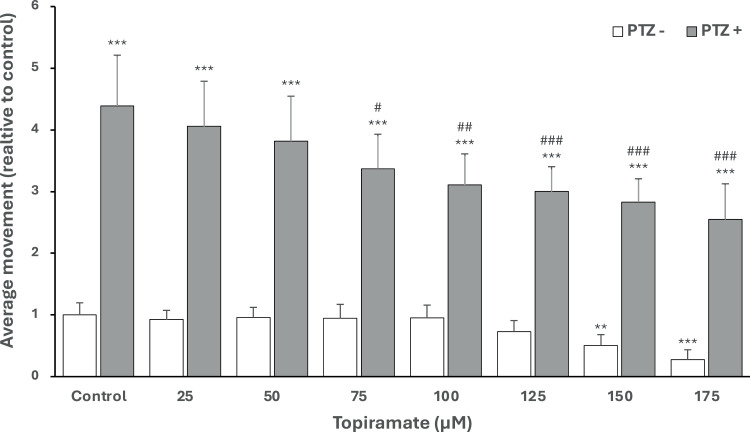
Effect of topiramate (TPM) on average larval locomotor movement without pentylenetetrazol (PTZ -), with PTZ-induced seizures (PTZ+ ); ** p < 0.01, *** p < 0.001, as compared to control (PTZ -); # p < 0.05, ## p < 0.01, ### p < 0.001 as compared to control (PTZ+); average movement is presented as referring to control (PTZ-); (1 = 29460 actinteg units).

Next, larvae were treated with TPM at a concentration of 25 to 175 μM. Only 150 μM and 175 μM concentrations decreased the locomotor activity of larvae compared to the control, as shown in Fig 4. TPM ( > 50 μM) significantly protected larvae against the PTZ. However, none of the dilutions were able to ameliorate the movements to the control level (Fig 5).

### The influence of CAF and TPM combination on larval locomotor activity

**Fig 6:**
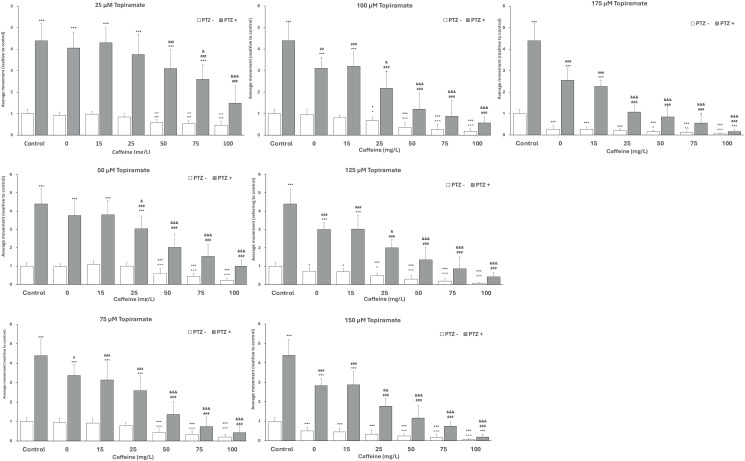
Effect of caffeine (CAF) and topiramate (TPM) combination on average larval locomotor movement; without pentylenetetrazol (PTZ -), with PTZ-induced seizures (PTZ + ); TPM concentration applied: 25, 50, 75, 100, 125, 150 and 175µM (graphs ordered in increasing TPM concentration); CAF concentration applied: 0, 15, 25, 50, 75, 100 mg/L (bars ordered in increasing CAF concentration in each graph); * p < 0.05, *** p < 0.001, as compared to control (PTZ-); # p < 0.05, ## p < 0.01, ### p < 0.001 as compared to control (PTZ+); ^ p < 0.05, ^^ p < 0.01, ^^^ p < 0.001, as compared to 0 CAF group (PTZ-); & p < 0.05, && p < 0.01, &&& p < 0.001, as compared to 0 CAF group (PTZ+); average movement is presented as referring to control (PTZ-); (1 = 29460 actinteg units).

Then, we investigated the effect of the combination of CAF and TPM on locomotor activity (Fig 6). When larvae were treated with 25 μM, 50 μM, or 75 μM of TPM with the addition of 50 mg/L, 75 mg/L, and 100 mg/L of CAF, their locomotor activity was significantly suppressed. In 100 μM TPM, also 25 mg/L CAF decreased the movement, while with higher TPM doses, all investigated CAF dilutions inhibited the movement (Fig 6).

In the PTZ model, when larvae were treated with 25 μM TPM, the addition of 50 mg/L, 75 mg/L, and 100 mg/L of CAF significantly suppressed the activity compared to the PTZ group, and zebrafish treated with 100mg/L CAF showed the activity at the level of control animals. With 50 μM, 75 μM, or 100 μM TPM, the 50 mg/L, 75 mg/L, and 100 mg/L CAF suppressed the movement to a control level. With higher TPM doses also, 25 mg/L CAF attenuated the activity to non PTZ treated animals. The combination of TPM in doses 75-175 μM and all investigated CAF dilutions suppressed the movement compared to the PTZ group (Fig 6).

Without PTZ, the addition of 15 mg/L CAF to TPM did not influence the larval activity, compared to TPM alone, in any investigated TPM dilution, while 50, 75, and 100 mg/L CAF suppressed it in every TPM concentration (Fig 6).

In the PTZ model, the addition of 15 mg/L CAF to TPM did not influence the larval activity, compared to TPM alone, in any investigated TPM dilution, while 50, 75, and 100 mg/L CAF suppressed it in TPM over 25μM (Fig 6).

## Discussion

This is the first study to characterize the influence of CAF and TPM in the PTZ-induced seizure model in zebrafish larvae on locomotor activity, as well as in drug combination. First, we evaluated mortality and HR in a larvae treated with CAF and TPM. Then, motility was assessed in both healthy and with induced epilepsy zebrafish. For both, we assessed a wide range of CAF doses, from 5 to 100 mg/L, and TPM doses, from 25 μM to 175 μM.

### The influence of CAF and TPM on HR

In our study, the control larvae at 5 dpf exhibit 155 BPM. CAF in dose 75 mg/L increased the larvae HR compared to the control group, while doses 175 mg/L and more decreased it. TPM does not affect the HR in any investigated doses. When combined, the BPM decreased when larvae were treated with 150 mg/L CAF and 200 μM TPM, 200 mg/L CAF and 200 μM TPM.

Maeda et al. presented that in 6 dpf larvae, HR was affected by CAF exposure in a dose-dependent manner At low concentrations (0.1 and 1 mg/L), it increased slightly compared to controls, but at concentrations ≥ 300 mg/L, HR decreased significantly. After 24 hours, bradycardia intensified, particularly at higher CAF doses (300–1000 mg/L). However, removing CAF after 4 hours allowed for partial HR recovery, with a full recovery observed at ≤ 500 mg/L and partial recovery at 1000 mg/L. CAF can cause bradycardia either via physiological mechanisms or toxicological effects [27]. As previously mentioned, the incidence of bradycardia and death in striped zebrafish may be utilized as a screening tool for drugs that prolong the QT interval and carry a risk of sudden death [28]. Rana et al. found that CAF concentrations between 1 and 25 mM inhibited heartbeat activity, resulting in the death of zebrafish embryos after about 2 to 3 dpf [29]. Zebrafish can significantly aid in studying pathophysiology and in large-scale screenings for drugs and toxins that could lead to fatalities, organ damage, and unusual behaviors. The evidence suggests that CAF has the potential to inhibit the hERG channel at concentrations between 1 and 5 mM, thereby inducing QT prolongation and increasing the risk of sudden cardiac death in individuals with existing conditions, such as polymorphic ventricular tachycardia [30].

While *in vitro* studies have indicated that TPM may have arrhythmogenic potential [31], evidence suggests that it does not elevate the risk of sudden, unexpected death caused by cardiac arrhythmia in epilepsy patients [32]. TPM does not increase the risk of major adverse cardiovascular events [33], but has been associated with decreased systolic and diastolic blood pressure among obese, diabetic patients, as assessed in a 32-week randomized clinical trial study of 69 subjects [34].

To date, no studies have assessed heart-related symptoms while taking CAF and TPM. The interplay between CAF and TPM revealed by this study aligns with the known pharmacological mechanisms of these agents and provides insights into their combined effects. CAF, a potent adenosine receptor antagonist, increases neuronal excitability and promotes excitatory neurotransmitter release, mirroring its ability to elevate HR at lower concentrations [35]. However, at higher concentrations, CAF’s toxicological effects, such as calcium overload and hERG channel inhibition, appear to induce bradycardia, reflecting disruptions in cellular homeostasis [35]. Conversely, TPM stabilizes neuronal activity through enhancement of GABAergic transmission, inhibition of AMPA/kainate glutamate receptors, and modulation of ion channels, contributing to its anticonvulsant efficacy [36,37]. The observed decrease in HR with combined CAF and TPM administration may indicate a dose-specific synergistic interaction, where CAF’s high-dose effects potentiate TPM’s membrane-stabilizing properties.

### The influence of CAF on larval locomotor activity

In our study, 50 mg/L, 75 mg/L, and 100 mg/L CAF decreased the average larvae movement. No statistically significant change in locomotor activity is observed at lower CAF concentrations, yet a slight increase in the activity is noticeable. In the PTZ-induced seizure model, the application of CAF in doses 50 mg/L, 75 mg/L, and 100 mg/L also resulted in a decrease of the average movement in comparison to the PTZ group, while zebrafish treated with 75mg/L and 100mg/L CAF showed the activity at the level of control animals.

In a study by Steenbergen et al., treatment of 6 dpf larvae with CAF (85mg/L) for 7 minutes followed by a washout did not affect total swim distance [38]. Conversely, a different study noted that a 4-hour exposure to the same concentration at 7 dpf significantly reduced travel distance, with effects more pronounced after 24 hours [39]. Confirmatory results were seen in larvae exposed for 2 hours to 10mg/L and 100mg/L of CAF, where the latter concentration significantly reduced swimming speed [22]. Additionally, exposure to CAF significantly decreased locomotor activity from 120 to 168 hours post-fertilization, particularly during dark periods [40]. In contrast, adult zebrafish injected with 10 mg/kg CAF exhibited increased locomotor activity [41]. At lower doses, CAF acts as a stimulant, but higher doses in adult zebrafish, as well as in mice and humans, have been shown to suppress locomotion and induce anxiogenic effects [42–46]. A study by Santos et al. indicated that while CAF concentrations below 5mg/L had no effect, levels between 10-25 mg/L increased, and doses above 50mg/L decreased locomotor activity compared to controls in adult zebrafish [47]. Egan et al. suggested that reduced activity at 100 mg/L might relate to predator avoidance behaviors such as freezing or hiding [42]. Further, exposure to higher doses (>50mg/L) increased freezing behaviors in adult *Danio rerio*, while lower concentrations did not affect this parameter [47]. Early exposure to CAF has also been reported by Chen et al. to cause muscle malformation in zebrafish, potentially affecting locomotor patterns [48]. Notably, exposure to 1.3 mM CAF in adult zebrafish induces a severe epileptic phenotype, and co-administration with subconvulsive doses of PTZ decreases the latency to the first seizure, suggesting a synergistic effect [19].

Our results align with those observed in the studies mentioned, showing that lower concentrations of CAF (5-25 mg/L) do not enhance locomotor activity, consistent with the findings at 10 mg/L CAF for 2 hours in 7 dpf larvae [22]. The same concentration increased activity in adults both when exposed for 2 hours and just prior to testing [41,47]. Similarly, 5 mg/L CAF did not impact adult zebrafish movement [47], a finding we presented. Our results, regarding suppressed locomotor activity in doses > 50 mg/L are also in line with other reports presenting reduced movement: 4 h and 24 h exposure (85mg/L) in 7 dpf, [39]; 2 h exposure (100 mg/L) in 7 dpf [22]; direct exposure before assessment > 50 mg/L in adult animals [47].

The results demonstrate that high doses of CAF (>50 mg/L) independently decrease larval movement in the PTZ seizure model, consistent with CAF’s dose-dependent effects on neural excitability and locomotor activity. At lower doses, CAF is known to increase neuronal activity and promote excitatory neurotransmission through adenosine receptor antagonism. However, at higher doses, toxicological effects such as calcium overload and disruptions in cellular signaling pathways likely contribute to reduced activity, as previously reported in similar models [29].

### The influence of TPM on larval locomotor activity

In our study, 150 μM and 175 μM TPM concentrations decrease the locomotor activity of larvae compared to the control. In the PTZ-induced seizure model, TPM ( > 50 μM) significantly protects larvae against the PTZ. However, none of the concentrations were able to ameliorate the movements to the control level.

Topiramate’s (TPM) effects on larval locomotor activity vary, with some studies showing a slight increase in total movement at 200 μM after 4 hours of exposure in 4 dpf larvae but no significant changes at lower concentrations or after longer exposure [49]. Exposure to TPM at 1, 3, and 10 mM for 24 hours in 6 dpf larvae showed no significant change in total distance in non-PTZ groups, yet it suppressed activity in the PTZ model [50]. TPM also inhibited seizure-like behavior in 7 dpf larvae after 18 hours of incubation at 200 μM but did not affect overall seizure duration in EEG assays [20]. A similar reduction in locomotor activity was observed in 7 dpf larvae after both 24 hours of incubation and acute exposure to 200 μM TPM [51]. In the PTZ-induced seizure model employing a lower dose of 10 mM PTZ over extended periods, both the TPM pretreatment and the acute exposure groups exhibited significantly reduced movement compared to controls. They used a lower PTZ dose (10mM) for a longer time, as higher PTZ concentrations can manufacture synaptic fatigue, exhaustion, and death, resulting in a reduction in swimming behavior in later time increments [51]. Unlike the findings of Afrikanova et al., who reported inconsistencies between locomotor and electroencephalogram assays [20], they demonstrated that TPM diminished both behavioral and neural activities, as evaluated through electroencephalogram and GCaMP studies[51].

In adult *Danio rerio*, TPM at doses of 20-30 mg/kg markedly reduced locomotor activity [52]. Several studies have observed that TPM doses ranging from 1 to 100 mg/kg did not alter spontaneous locomotor activity in both mice and rats [53,54], even when administrated for 45 days at a dose of 100 mg/kg in rats [55]. Conversely, Alaverdashvili et al. found that non-toxic doses of TPM increased locomotor behavior [56]. Additionally, a slight yet not significant reduction in rat locomotion was observed with oral administration of TPM [57]. The effects of TPM on seizure behavior in equivalent rodent PTZ assays have been mixed. While seizure suppression at the maximum tolerated dose in mice has been reported, such effects were not observed in rats [58], and some studies reported no activity at all [59,60].

Our study highlights the efficacy of TPM in protecting zebrafish larvae from the effects of PTZ-induced seizures, with concentrations exceeding 50 μM providing significant protection. However, the activity levels in these larvae did not return to those observed in the control group, aligning with findings reported for 7 days post-fertilization (dpf) larvae exposed to 180 μM TPM for 18 hours [61]. This partial protection suggests that TPM, while effective in mitigating hyperexcitability, does not fully restore normal neuronal and behavioral states under certain exposure durations and concentrations. Interestingly, extending the exposure period to 24 hours at a higher dose of 200 μM TPM resulted in activity levels comparable to controls in 7 dpf larvae [2]. This observation may reflect a dose- and time-dependent enhancement of TPM’s pharmacological effects, including its ability to modulate multiple pathways involved in neuronal stabilization.

In our research, TPM concentrations of 150 μM and 175 μM led to decreased locomotor activity in larvae compared to controls, aligning with results seen in 7 dpf larvae exposed to 200 μM for 24 hours [51] but diverging from findings that showed increased movement at the same concentration and exposure duration in 4 dpf larvae [49]. Notably, the latter study also found no differences at a concentration of 100 μM, a result we observed in our experiments [49]. Furthermore, we observed that TPM concentrations greater than 50 μM effectively protected larvae against PTZ-induced effects, though they did not reduce activity to control levels, consistent with observations in 7 dpf larvae exposed to 180 μM for 18 hours [20]. However, in 7 dpf larvae, a 24-hour exposure to 200 μM TPM significantly lowered activity to control levels [51].

The differential outcomes observed with varying TPM concentrations and exposure times underscore the complexity of its pharmacodynamic profile. While shorter exposures to lower concentrations may alleviate seizure activity, higher doses or prolonged exposures appear necessary to fully normalize behavior. These findings suggest that the pharmacokinetics of TPM, including its absorption, distribution, and sustained action at target sites, play a critical role in its effectiveness. As we know, the zebrafish model differs from human drug pharmacokinetics and studies on this model should be carefully reviewed to facilitate human.

### The influence of CAF and TPM combination on larval locomotor activity

In our study, in the PTZ-induced seizure model, when larvae were treated with 25 μM TPM, the addition of 50 - 100 mg/L of CAF significantly suppressed the activity compared to the PTZ group, and zebrafish treated with 100mg/L CAF showed the activity at the level of control animals. With 50 - 100 μM TPM, the 50 - 100 mg/L CAF suppressed the movement to a control level. With higher TPM doses also, 25 mg/L CAF attenuated the activity to non PTZ treated animals. The combination of TPM in doses 75-175 μM and all investigated CAF dilutions suppressed the movement compared to the PTZ group. The addition of 15 mg/L CAF to TPM did not influence the larval activity, compared to TPM alone, in any investigated TPM dilution, while 50-100 mg/L CAF suppressed it in TPM over 25μM.

Research into the interactions between CAF and TPM is relatively sparse. When CAF and TPM were co-administered, the interaction was dose-dependent. At low CAF doses (15 mg/L), no significant alteration in locomotor activity was observed, suggesting minimal interference with TPM’s protective effects. However, higher CAF doses (>25 mg/L) markedly reduced larval activity, even in the presence of TPM. This could indicate a synergistic toxicity or an antagonistic interaction that overrides TPM’s stabilizing effects. Studies have shown that higher doses of CAF (23.1 mg/kg and 46.2 mg/kg), administered either acutely or chronically, can increase the effective dose of TPM necessary to protect 50% of mice (ED50) from seizures induced by maximal electroshock (MES) [62]. In contrast, lower doses of CAF (5.7 and 11.5 mg/kg) did not influence the anticonvulsant effects of TPM [62]. Similar results were observed in rats, where CAF significantly raised the ED50 value for TPM in MES-induced seizures [7]. A single high dose of CAF seems more likely to trigger seizures than repeated low doses of CAF, suggesting the development of tolerance to A1- and A2A- receptor blockade by CAF [5]. In studies with adult zebrafish using a suboptimal concentration (4 mM) of PTZ, CAF was shown to decrease seizure latency in a dose-dependent manner [63].

Our findings indicate that adding 15 mg/L CAF to TPM did not affect larval activity at any investigated TPM concentration, aligning with the effects of lower CAF doses in mice [62]. However, unlike observations in mice [62] and rats [7], higher doses of CAF in our study did not increase the TPM amount required to protect against PTZ. We observed that CAF concentrations above 25 mg/L significantly reduced locomotor activity more than TPM alone, with more pronounced effects at higher CAF concentrations.

The reasons for reduced locomotion in larvae treated with combinations of CAF, TPM, or PTZ remain unclear. TPM is known to have multiple potential mechanisms of action [64] with paresthesia noted as a side effect [65]. Thus, the potential for “on/off-target” effects of TPM, particularly in combination with PTZ in developing zebrafish larvae, should not be disregarded. TPM has also been shown to exert an anxiolytic-like effect in adult *Danio rerio* and rats [52,57]. In the literature, terms like immobility and freezing are often used interchangeably, although immobility can be induced by sedative drugs, resulting in hyperlocomotion, akinesia, or paralysis [66]. Clinical studies also list sedation as a side effect of TPM [67], with both immobility and freezing characterized by a complete cessation of movement except for gill and eye movements. Stress-induced freezing frequently involves rapid opercular movements [66].

CAF primarily acts by antagonizing the four adenosine receptor subtypes, but its mechanisms also involve calcium channels, phosphodiesterases, GABA-A receptors, and phosphatidylinositol-3-kinase [39].

TPM’s mechanisms of action include enhancing GABA-mediated inhibition, reducing glutamatergic neurotransmission via AMPA/KA receptors, and inhibiting voltage-operated sodium and L-calcium channels [68]. The exact mechanisms by which TPM affects locomotor activity in rodents and fish remain underexplored, and further research is needed to determine how species-specific differences may influence these effects [52]. Wijk et al. highlight that by studying the formation and elimination of drug metabolites in zebrafish, researchers can better predict how drugs will behave in higher vertebrates, including humans. This is particularly important for drug development and safety assessments [69].

The primary mechanism of pentylenetetrazol (PTZ) is thought to inhibit GABA activity at the GABA-A receptor, although several non-GABAergic drugs also effectively prevent PTZ-induced seizures in *Danio rerio* [70]. This broad effectiveness renders the zebrafish PTZ screen less specific compared to mouse and rat models, where PTZ seizures specifically identify GABA-targeting anticonvulsants.

Recent studies indicate that CAF may cause epigenetic changes that affect neuronal excitability, potentially underlying epilepsy [71]. Sleep deprivation is a known trigger for seizures, and epilepsy itself can disrupt sleep patterns. Given that CAF counteracts fatigue and blocks the sleep-promoting effects of adenosine, its impact on seizure susceptibility may be linked to its ability to disturb sleep. Conversely, reduced sleep due to seizures may increase CAF consumption, thereby heightening seizure risk and creating a self-perpetuating cycle [5].

The translation of findings from animal models to humans poses significant challenges due to differences in CAF metabolism between species and even within the same species. The doses of CAF used in animal research are typically much higher than the average human consumption. In humans, CAF plasma concentrations ≥ 15 mg/L generally stimulate the central nervous system and heart, while overdose effects at concentrations of 190 mg/L and lethal toxicities at 350 mg/L to 567 mg/L have been documented [72]. Although no clinical studies have conclusively confirmed the provocative effects of CAF on seizure susceptibility observed in animal models, these studies often rely on self-reporting, leaving room for response and selection biases [5].

In conclusion, the interaction between CAF and TPM can be interpreted in two distinct ways. First, the consistent levels of TPM in the presence of CAF suggest that CAF may counteract the anticonvulsant properties of TPM [7]. Alternatively, CAF may reduce seizure latency, indirectly increasing the required dose of TPM, similar to other seizure precipitants [63]. The observed dose-specific interactions between CAF and TPM emphasize the importance of careful dose optimization when considering drug combinations. While TPM effectively mitigates seizure activity, its efficacy may be compromised at higher CAF doses, necessitating further investigation into the mechanistic basis of this interplay. The study provides valuable insights into the interactions between CAF and TPM, which may inform future research on human epilepsy. However, the extrapolation of these results to other species should be approached cautiously due to physiological differences.

### Study limitations

The above-mentioned studies show contradictory results, which may arise from the use of different models referring to different epilepsy types, various ways of inducing seizures, various methods for drug administration, different doses of chemical substances, alternative measurement devices, acquiring methods, and assessed parameters. It also should be noted that larvae behavior varies depending on the time of day or light [25,26]. The limitation of our study is also the fact that we have used only one seizure indicator.

## Supporting information

S1 Data Raw data. (XLSX)
